# Berberis in Colic with Inflammatory Stricture

**Published:** 1882-12

**Authors:** G. N. Birgham

**Affiliations:** Grand Rapids


### BERBERIS IN COLIC WITH INFLAMMATORY STRICTURE.

Case began with pain in the right lumbar region between the kidney and rim of the ilium, extending downward somewhat into the pelvis. The pain was of an *aching, tensive* character, the sensation of *fixity*, or being confined by unyielding bands, was noticeable. This fixed pain continued for hours in a more or less severe manner, relaxing and then returning, giving a very uncomfortable feeling most of the time, and suspicious from its very fixedness. Not enough trouble and concern was experienced, however, to prevent continued labor, though motion increased the unpleasant sensations. It was a very hot day on which the attack came on and cold water had been partaken of quite freely. A little before sundown the pain increased and there was a desire for stool and a free passage. No relief followed; rather, the pains became more violent and soon almost unendurable. Being from home, we drove for two miles rapidly and immediately went to bed utterly prostrated. Took Nux and a hot-water enemata with no relief, and as the extremities were very cold, followed with Veratrum, which did little or no good. Being without the benefit of homœopathic counsel and fearing hernial stricture or interseption, called a friend of another school till better help could be obtained, as we had to send six miles for one of our own school. Diagnosis somewhat uncertain, but case very alarming in character. A very warm enemation, with thirty drops of laudanum given. A mitigation of sufferings for about an hour, when they returned with more violence than ever. We were in a collapsed state from the intensity of pain, covered with beads of cold sweat all over, nose pinched and countenance shrunken. Veratrum was continued but we did not perceive any particular effect. The last paroxysm seemed to come in three great waves, at which time there was the sensation of *nerve-stretching* and breaking apart of portions of the cord. The same fixed, tense, unyielding pain kept up all the time in the region of the right kidney and a little below. By force of will we ran over some leading symptoms in our repertory and came upon one or two under Berberis which best met our feelings, and commenced taking it. To our great satisfaction, the pains in the loins and under the rim of the pelvis for the first time began to yield. We have little doubt that Berberis saved our life. The movement of the bowel was probably below the stricture, for nothing passed the anus from that time for five days, except when, by much straining, the water used as an enema was forced away in a small stream as clear water. Not even a particle of gas escaped. The sensation was that the intestines *were stiff* and powerless. Berberis controlled, however, all untoward symptoms, and by keeping in bed curative reaction continued and the cure was completed by adose of sulphur$^{20m}$ (Fincke), noisy flatulence following soon after its use. The case was the most violent I ever met and was by counsel considered hopeless. The lumbar symptoms are the most important to note. The case, to us, was valuable not only in saving life, but in demonstrating the value of our law of cure. We knew little or nothing of the remedy before—nothing clinically—but the proving had given us the key.

*Grand Rapids.* G. N. Brigham, M. D.

